# Crystallographic Lattice Boltzmann Method

**DOI:** 10.1038/srep27172

**Published:** 2016-06-01

**Authors:** Manjusha Namburi, Siddharth Krithivasan, Santosh Ansumali

**Affiliations:** 1 Jawaharlal Nehru Centre for Advanced Scientific Research, Jakkur, Bangalore, 560064, India

## Abstract

Current approaches to Direct Numerical Simulation (DNS) are computationally quite expensive for most realistic scientific and engineering applications of Fluid Dynamics such as automobiles or atmospheric flows. The Lattice Boltzmann Method (LBM), with its simplified kinetic descriptions, has emerged as an important tool for simulating hydrodynamics. In a heterogeneous computing environment, it is often preferred due to its flexibility and better parallel scaling. However, direct simulation of realistic applications, without the use of turbulence models, remains a distant dream even with highly efficient methods such as LBM. In LBM, a fictitious lattice with suitable isotropy in the velocity space is considered to recover Navier-Stokes hydrodynamics in macroscopic limit. The same lattice is mapped onto a cartesian grid for spatial discretization of the kinetic equation. In this paper, we present an inverted argument of the LBM, by making spatial discretization as the central theme. We argue that the optimal spatial discretization for LBM is a Body Centered Cubic (BCC) arrangement of grid points. We illustrate an order-of-magnitude gain in efficiency for LBM and thus a significant progress towards feasibility of DNS for realistic flows.

Fluid dynamics simulations are an integral part of science and engineering applications. Such simulations are parameterized by a single non-dimensional number — known as Reynolds number Re — which ranges from ~10^−3^ for many biological applications to ~10^9^ for atmospheric flows. The size of the grid required to resolve all length-scales to perform DNS is often too high for realistic applications^1^. In practice, applications of Computational Fluid Dynamics (CFD) to the physics of complex flows in the context of fluid-structure interaction often requires highly accurate and adaptive methods for complex geometries. Currently, it is widely accepted that DNS of separated turbulent flows will be feasible only after a decade^2^,^3^,^4^. Indeed, recent studies have advocated the use of a hybrid approach where both Large Eddy Simulation (LES) and Reynolds Averaged Navier-Stokes (RANS) are used in appropriate parts of the domain^2^,^3^. Similarly for many biological applications — where one needs to perform simulation of suspensions — the resolution of near-field hydrodynamics for particles requires an exorbitant number of grid points for DNS. Thus an algorithmic improvement which can make DNS feasible and realistic for hydrodynamic simulation is critically required.

For DNS of homogeneous turbulence, the most accepted methodology is Pseudo-Spectral (PS) method, which has spectral accuracy unmatched by alternative methods. However, it has relatively poor parallel scaling and is largely limited to periodic domains and to a few other simple geometries such as channel flow^5^. Thus, in the last two decades, a number of algorithms such as LBM were developed for accelerating hydrodynamic simulations. LBM, with its simplified kinetic picture on a lattice is easily parallelizable and scalable. It is therefore an alternate numerical method for applications as wide ranging as fluid turbulence, polymer dynamics, density functional theory, soft matter etc.^4^,^6^,^7^,^8^,^9^,^10^,^11^,^12^,^13^,^14^,^15^,^16^,^17^,^18^,^19^,^20^. Often it is argued that kinetic descriptions are inherently better suited for both complex flow phenomena such as turbulence and for modeling complex flow physics^11^,^12^,^21^,^22^. In contrast to the pseudo-spectral method however, lower order models such as LBM often require more than twice number of grid points in every direction and thus, they require an order-of-magnitude (2 to 2.5 times in each direction) more number of grid points in 3D for the same accuracy. Nevertheless, in practice, LBM is competitive with respect to PS due to very high parallel efficiency and flexibility in handling complex geometries. Indeed, in a heterogeneous computing environment, LBM and PS are able to perform DNS at comparable Reynolds Numbers for simple flows^4^,^23^. Thus similar to conventional CFD, one often needs to use explicit turbulence models for simulating relevant high Re applications^6^,^24^. Thus to simulate realistic turbulent flows without any explicit turbulence model is far-fetched even with efficient methods such as LBM. Similarly to study the collective dynamics of particles at low Reynolds Numbers, one needs to introduce point-particle like models due to resolution constraints. For instance, even to resolve a simple shape such as a sphere at these Reynolds Numbers, ~20^3^ grid points are required.

Thus improving the accuracy of LBM, while keeping its parallel efficiency and large time-stepping intact, is an important challenge. The only working methodology for improving the accuracy of LBM is to refine the grid near the solid body or in zones of extreme flow variations^25^,^26^. A fundamental problem with such approaches is that the local accuracy of the method remains unchanged and optimization is done only with respect to the global distribution of grid points. For example, for decaying turbulence in a periodic geometry, local grid refinements cannot improve the accuracy of LBM.

Before describing our new method, we briefly summarize basic LBM algorithm. In any *M*-dimensional model (with *M* being 1, 2 or 3)^20^,^27^, typically denoted by *DMQN*, one defines a set of *N* discrete velocities *c*_*i*_, 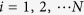. To each discrete velocity a weight *w*_*i*_ > 0 is assigned such that 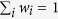.

The central quantity of interest in the method is the discrete population *f*_*i*_(**x**, *t*), at the location **x** and time *t*. In D-dimensions, macroscopic quantities such as mass density *ρ*, momentum density *ρ***u** and energy density *E* ≡ (*ρu*^2^ + *Dρθ*)/2, with reduced temperature *θ* are,


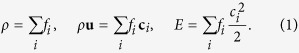


Other higher order moments of physical interest are the pressure tensor *P*_*αβ*_ and the third order moment *Q*_*αβγ*_ defined as,


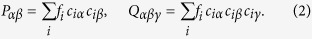


The non-equilibrium part of the pressure tensor is the stress tensor and the trace *q*_*α*_ ≡ *Q*_*αγγ*_ of the third order moment is related to the heat flux.

An important and relevant example of LBM is provided by the D3Q27 model for which the set of discrete velocities and the corresponding weights are given in Table 1.

Here we note that for standard LBM using the D2Q9 or the D3Q27 model, the lattice introduces an artificial closure,


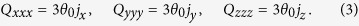


The evolution equation for the populations *f*_*i*_ with the Bhatnagar-Gross-Krook (BGK) model collision term is^28^,


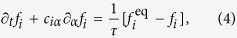


where the discrete equilibrium 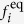 is typically written as^20^,


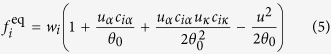


with *θ*_0_ as the reference temperature and on any given lattice the weights satisfy the conditions,


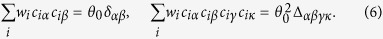


These constraints on weights ensure that


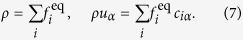


For such models, the higher order moments are


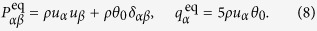


## Computational Method: Crystallographic Lattice Boltzmann Model

**Figure 1:**
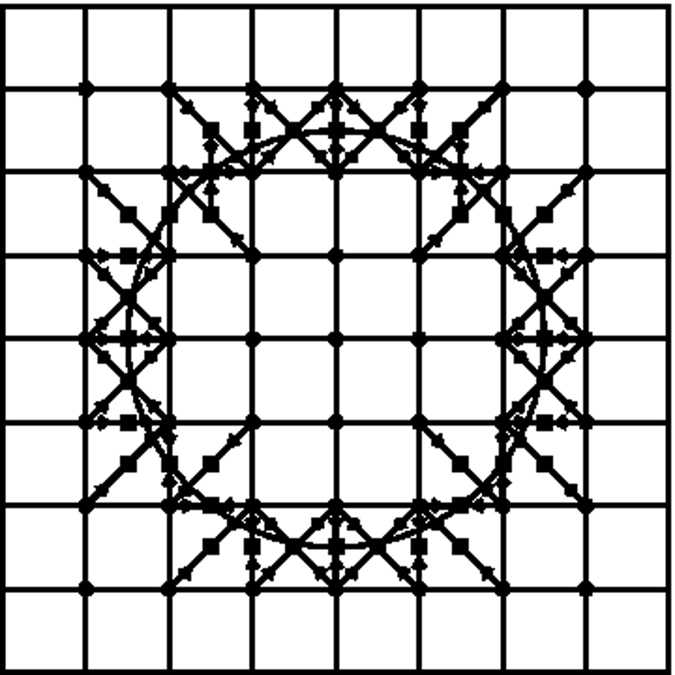
The boundary points are located at the half distance between the lattice points. Here, the boundary points are shown by solid squares and lattice points by solid circles.

**Figure 2:**
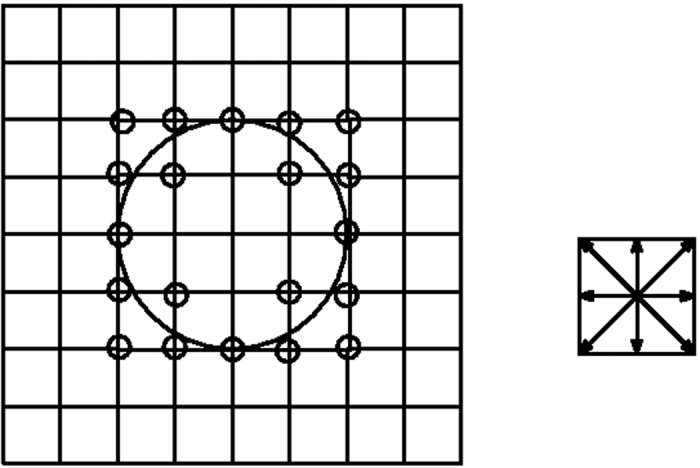
Left: grid points near the boundary on the SC grid. Right: Links on the SC grid.

**Figure 3:**
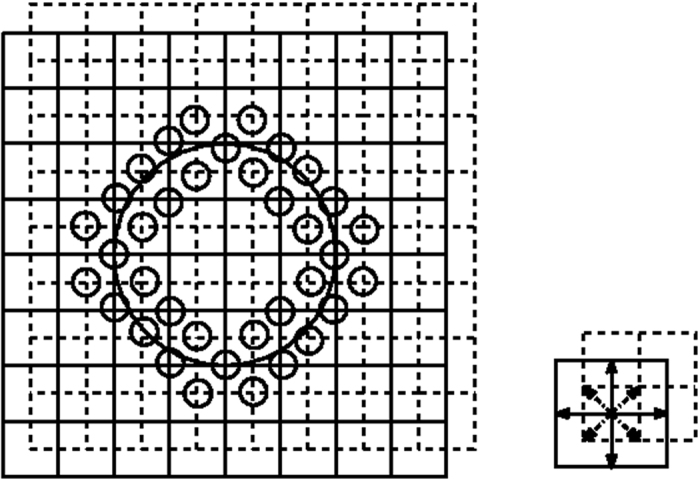
Left: grid points near the boundary on the BCC grid. Right: Links on the BCC grid.

In this section, we introduce our new grid structure which drastically improves the performance of LBM without compromising its on-lattice streaming and other computational efficiencies. We begin with the observation that the introduction of the stair-case geometry, as done in LBM, is similar to the generation of a Wigner-Seitz cell for a given lattice structure^29^. This is pictorially shown in Fig. 1. With this point of view, it becomes apparent that, out of all possible space-filling arrangements, the Simple Cubic (SC) Lattice for space discretization used by LBM is not very efficient for representing local curvatures. Intuitively, one would expect an octahedron to be a better object in resolving local curvatures. Thus, a better choice for spatial grid distribution is a Body-Centered Cubic (BCC) arrangement of grid points for which the Wigner-Seitz cell is a truncated octahedron^30^. To illustrate this point, we consider a rhombic grid in 2D. Figures 2 and 3 show that the discrete approximation of a circle is more accurate on the BCC grid than on an SC grid: the total number of boundary points are almost double.

For any given lattice, the links on the grid in Fig. 3 act as discrete velocities in LBM. It is also clear from Fig. 3 that, unlike previous attempts to design body-fitted grids, with this lattice, we achieve better space coverage and also a unification of the discretization of space and velocities.

Based on this insight, we invert the argument of LBM and make spatial discretization the central point and decide the velocity space based on this discretization. We generalize the D3Q27 model on a BCC lattice by changing velocity vectors of type {±1, ±1, ±1} to {±1/2, ±1/2, ±1/2}. Hereafter, this model will be referred as the RD3Q27 model. The set of discrete velocities and corresponding weights are listed in Table 2. For the RD3Q27 lattice, the reference temperature changes to *θ*_0_ = 1/5 (see Eq. (6)). This procedure also removes some of the known artifacts associated with the velocity space discretization used in LBM. Finally, we demonstrate that these modifications improve the simulation efficiency by more than an order-of-magnitude. An alternate view point of the method is provided by entropic construction of LBM. In this formulation, one begins with a given *H*-function^31^,^32^,^33^ and construct equilibrium as the minimum of the *H*-function under constraints. Typically, one constructs an isothermal equilibrium, where energy conservation is ignored. It can be shown that the true entropic equilibrium is well approximated by Eq. (5) at least till order O(Ma^2^), where Ma is the Mach number. In dimensions higher than one, it is also possible to create the energy conserving equilibrium which we will use later. On the RD3Q27 lattice, following Ansumali *et al*.^33^, we choose to construct the energy conserving equilibrium as the minimizer of the *H*-function. The choice of the energy conserving equilibrium is largely dictated by the increased stability of those models^34^,^35^. The explicit expression for the energy conserving equilibrium distribution function is:


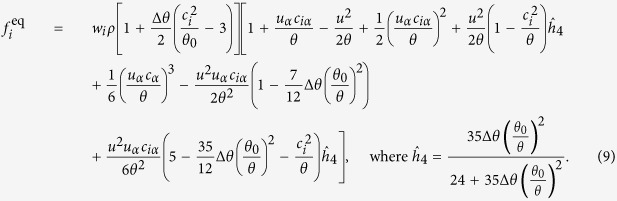


This ensures the conservation of density (*ρ*), momentum density (*ρu*_*α*_) and energy density (*ρu*^2^ + 3*ρθ*) during collision. The conserved quantities in terms of the equilibrium distribution function can be written as:


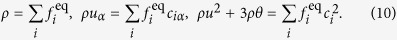


At *θ* = *θ*_0_, the explicit expression for the equilibrium distribution function is:


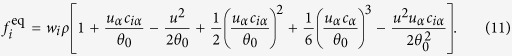


The corresponding equilibrium stress tensor and heat flux vector are given by,


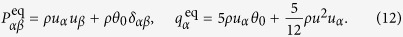


The better representation of extended hydrodynamics by present model can be seen by writing discrete moment chain. This moment chain is very similar to Grad’s equation of the form^12^,^36^,^37^:


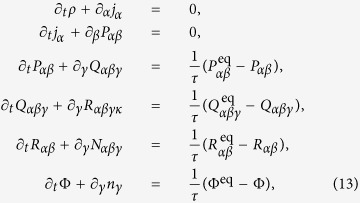


where 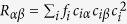 and 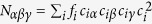. The closure relations for the higher order moments *R*_*αβγκ*_ and *N*_*αβγ*_ written in terms of other moments are given in Supplementary Appendix A. For the present model, *Q*_*xxx*_, *Q*_*yyy*_, *Q*_*zzz*_ are independent variables and artificial closures as shown in the previous section (Eq. 3) do not exist. This happens because, in the diagonal direction |*c*_*ix*_| is 1/2 rather than 1. Thus one can expect that RD3Q27 should show enhanced accuracy for finite Knudsen flows as compared to D3Q27^38^. This set-up provides a good indication of the convergence of the discrete velocity model towards the Boltzmann equation. Finally, following standard route of discretization along characteristic directions using trapezoidal rule^20^, we can write fully discrete version of the kinetic Eq. 4 as


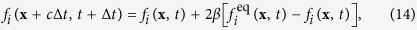


where *β* is the discrete relaxation time.

Notice this is similar to D3Q27 model, if we choose Δ*t* = Δ*x*, the advection happens from one lattice point to another and thus the method does not require any spatial interpolations. We recall that the BCC grid in 3D has an alternate interpretation in terms of replica of simple cubic grids displaced from each other by Δ*x*/2 in each direction where Δ*x* is the grid spacing.

## Results

**Figure 4:**
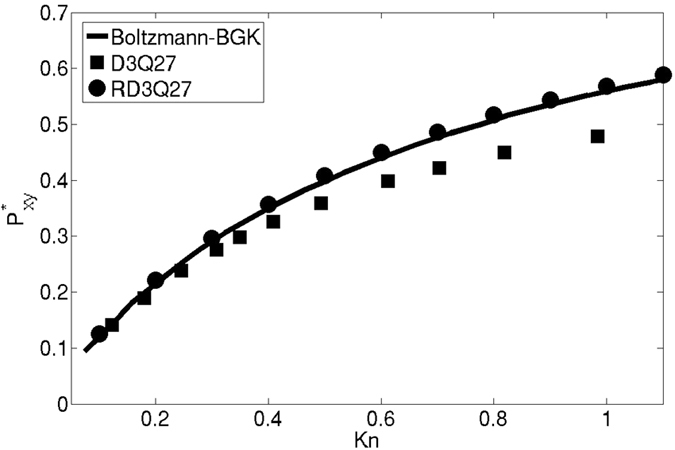
Comparison of shear stress profile for Couette flow configuration using D3Q27, RD3Q27 with Boltzmann-BGK model.

In this section, we present the results for standard canonical problems using the current RD3Q27 model. We first consider the planar Couette flow, a test problem for investigating the convergence of discrete velocity models towards the solution of the continuum Boltzmann-BGK equation^28^. In this set-up, the fluid is enclosed between two parallel plates separated by a distance L. The bottom plate at y = −L/2 moves with a velocity *U*_1_ and top plate at y = L/2 moves with a velocity *U*_2_. For such boundary value problems relevant to micro-flows, one uses the discrete version of diffused wall approximation in LBM^12^. For this set-up, it is known that the D3Q27 model has poor accuracy at finite Knudsen numbers (defined as 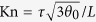); it works reasonably well only for Kn < 0.1. It can be seen in Fig. 4, the results of the RD3Q27 and D3Q27 models are similar at low Kn (<0.1). Further, at high Kn, the shear stress 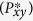 predicted by RD3Q27 is in close agreement with the continuum Boltzmann-BGK solution.

The solution for the dimensionless shear stress obtained by solving the moment chain for the RD3Q27 model is,


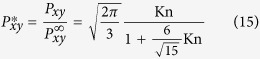


where 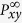 is the shear stress at Kn → ∞ of the Boltzmann-BGK model and is given by


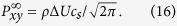


It is evident that removing the artifact on the third moment has led to better agreement with the Boltzmann BGK values. Thus, unlike D3Q27 model, accuracy for the flows at finite Knudsen number has improved drastically. However unlike higher order LBM, kinetic boundary layer is not captured by the method.

To demonstrate the efficiency of this new framework for turbulence, we chose the setup of decaying turbulence starting with the Kida vortex initial condition^4^,^23^. In this flow, starting with a smooth initial condition, the enstrophy (Ω) and the maximum vorticity show very rapid growth. Thus it is not surprising that, for this set-up, the standard D3Q27 lattice Boltzmann model fails to make accurate predictions unless the number of grid points per direction is more than twice those for an equivalent spectral simulation.

**Figure 5:**
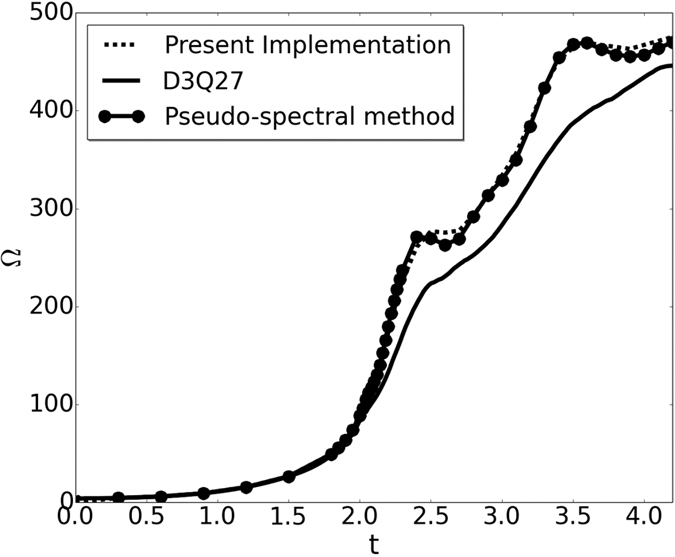
Variation of Enstrophy (Ω) with time (t) for Kida-Pelz flow at Re = 10,000 on 1200^3^ grid.

In Fig. 5, the performance of our new BCC lattice based RD3Q27 velocity model is contrasted with the pseudo-spectral and the D3Q27 model for Re = 10,000. In the present model, Re is defined as, *ρU*_0_/*ν*, where in the PS method Re = 1/*ν* as *U*_0_ = 1. It is evident from the results shown in Fig. 5 that our new method has drastically improved the performance of standard LBM. This gain can be understood in terms of the spectral representation of a bandwidth-limited function (i.e. whose Fourier coefficients are zero above a cut-off wave-number). Essentially, for a fully resolved three-dimensional simulation in a periodic geometry, a reasonable approximation is to assume that the functions to be modeled are isotropic and the resolution is bandwidth limited. This implies that, in Fourier space, there is no preferred direction and, hence, the efficient distribution of grid points is equivalent to the sphere-packing problem^30^,^39^. Thus the most efficient way to distribute points in the Fourier space is to arrange them as a FCC lattice. Consequently the best sampling lattice in real space is the BCC lattice, which is the dual of the FCC lattice^30^,^39^ in the Fourier space. Thus the numerical gain in simulation accuracy is associated with the efficient choice of the lattice.

It must be noted that the parallel and serial efficiency of this new model is very similar to the standard D3Q27 model. Thus for the first time, LB simulations are able to surpass higher order models such as the spectral method. Here we emphasize that this new route to increase accuracy is not an alternative to building higher order LBM for better accuracy^23^. Indeed, by constructing higher order LB on BCC, the accuracy can be further improved.

Although RD3Q27 uses a grid of size 2*N*^3^ as compared to standard case of *N*^3^, the performance gain is almost an order-of-magnitude. This happens because, for the same accuracy of simulation, the usual D3Q27 model requires a grid size of (*MN*)^3^, where M ranges from 2–2.5. This implies that the saving in the number of grid points is in the range of (*MN*)^3^/2(*N*)^3^, which is 4–7.8 times. Hence the saving in time is (*MN*)^4^/2(*N*)^4^ i.e., at least 8 times (on a coarser grid, the time steps are larger).

**Figure 6:**
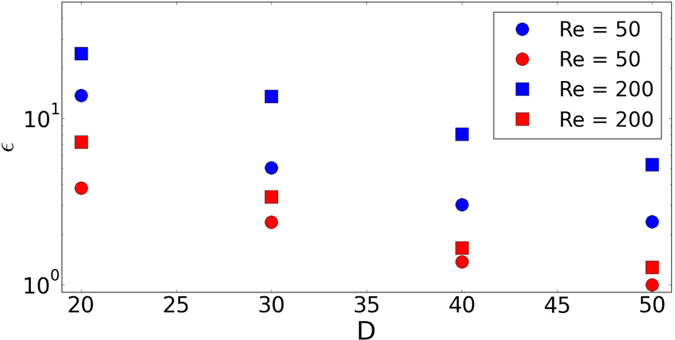
Comparison of percentage error (*ε*) in the mean value of *C*_*D*_ with increasing diameter (in lattice points) for flow past sphere between D3Q27 (in blue colour) and RD3Q27 (in red colour) at Re = 50 and 200.

**Figure 7:**
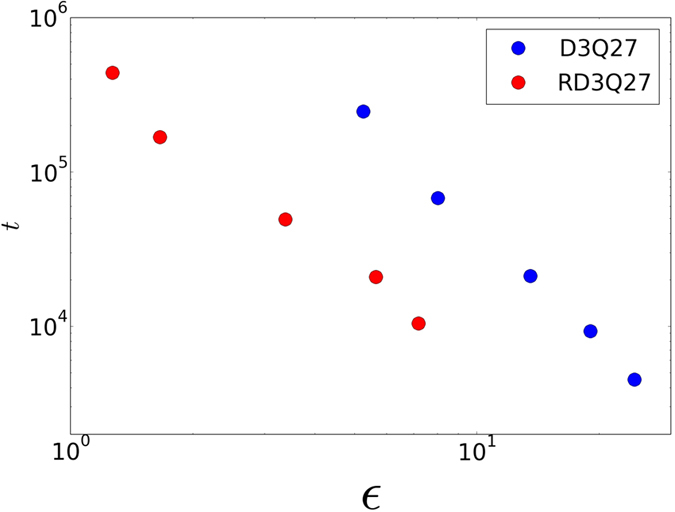
Simulation cost (t in seconds) for one convection with respect to the percentage error (*ε*) for flow past sphere at Re = 200. Simulations were performed for one convection time (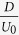, where *U*_0_ is the free stream velocity) on an Intel Xeon based system (CPU Model: E5-2670) with 16 cores.

To demonstrate this gain in the capability of LBM, let us consider the classical problem of flow past a sphere^40^,^41^,^42^,^43^. The simulations were performed for two different values of Re with Ma = 0.05 for D3Q27 and RD3Q27 models. The domain size, in terms of diameter *D* (measured in lattice unit), of 16*D* × 8*D* × 8*D* was used for Re = 50 and 20*D* × 10*D* × 10*D* for Re = 200. The simulations were performed on an Intel Xeon based system (CPU Model: E5-2670) with 16 cores. Figure 6 shows the percentage error (*ε*) in the mean value of 
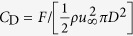
 from the experimental value of *C*_D_ = 1.59 for Re = 50 and *C*_D_ = 0.78 for Re = 200^42^. From this figure, it is evident that RD3Q27 performs an order-of-magnitude better than D3Q27. This gain in performance can be seen more conveniently in Fig. 7, where percentage error (*ε*) is plotted against CPU-time(t in seconds). In this plot, one can see that for the same error, present method requires an order-of-magnitude less time when compared to D3Q27 LBM.

**Figure 8:**
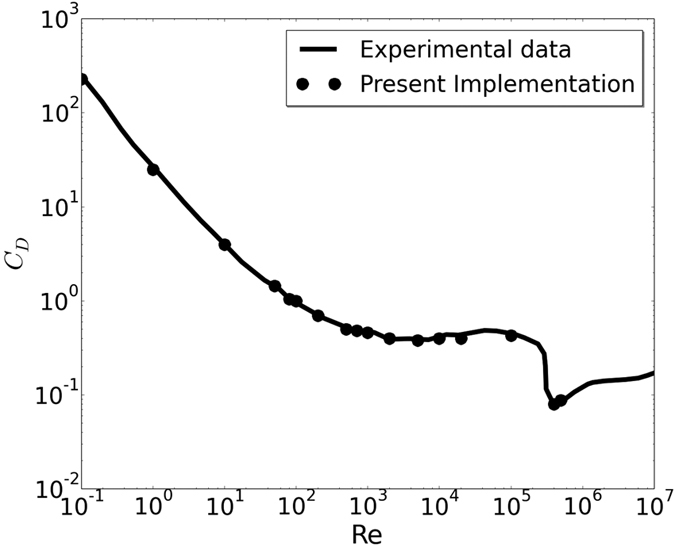
Variation of drag coefficient (*C*_*D*_) with Re for flow past a sphere compared with experimental data^40^.

**Figure 9:**
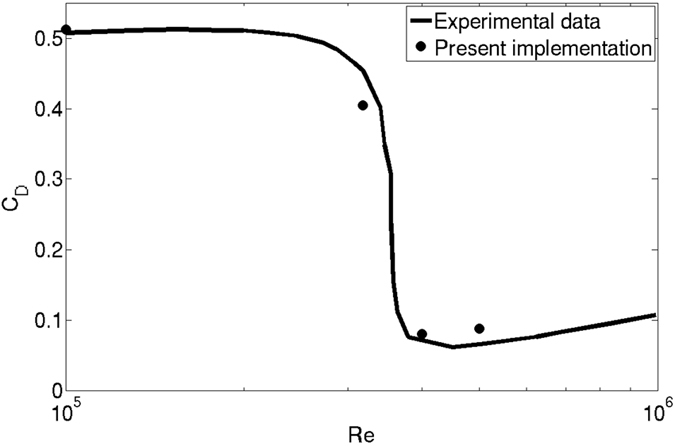
Drag coefficient (*C*_*D*_) zoomed into the range of Re = 10^5^ to 10^6^ with linear scaling.

**Figure 10:**
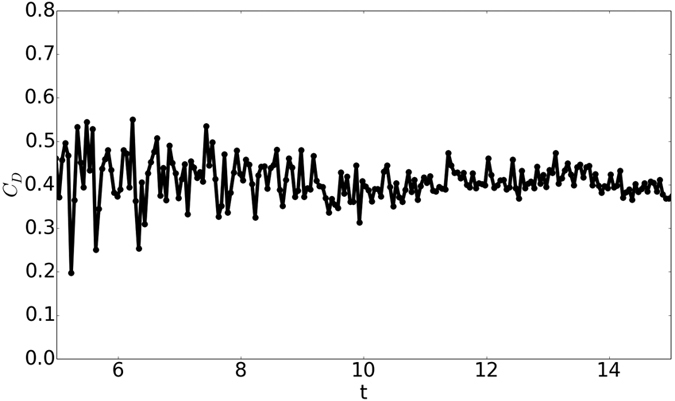
Variation of the drag coefficient (*C*_*D*_) with time for the flow past sphere at Re = 3.18 × 10^5^.

**Figure 11:**
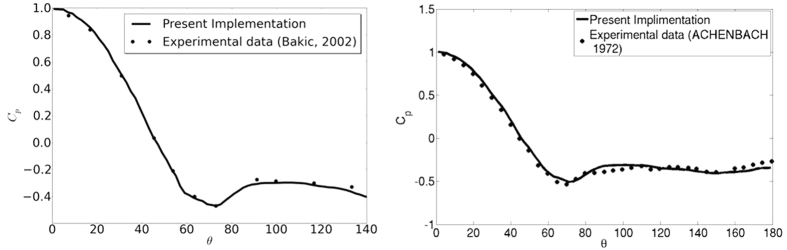
Distribution of pressure coefficient (*C*_*P*_) on the surface of the sphere at Re = 10^5^ (left) and Re = 1.62 × 10^5^ (right).

**Figure 12:**
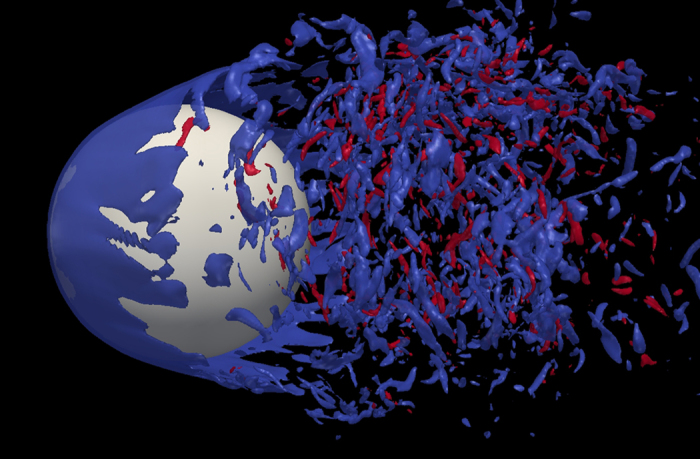
The snapshot of *ω*_*ϕ*_ for flow past sphere at Re = 10^5^.

As the result for Re = 200 suggests that the computing requirement for present model is substantially lower than LBM, we performed this simulation of flow past sphere for a wide range of Reynolds numbers. To the best of our knowledge, none of the existing methods can predict the drag behavior over the wide range of Reynolds Numbers for which experimental data is available. In Figs 8 and 9 the drag coefficient *C*_D_, predicted by present model is contrasted with the experimental result in Achenbach *et al*.^40^ and Bakic *et al*.^44^ with Re = *UD*/*ν* where *D* and U are the diameter and speed of the sphere in lattice units. The values of *C*_D_ for Re = 10^5^ and 3.18 × 10^5^ are depicted in Table 3. Figure 10 shows the variation of drag coefficient *C*_D_ with time at Re = 3.18 × 10^5^. We have implemented a version of the diffuse bounce-back rule as described in Krithivasan *et al*.^45^, for the solid-fluid interface at the body. For inlet-outlet, we have used Grad’s closure approximation as described in Chikatamarla *et al*.^46^. To highlight the reliability and robustness of our approach, the angular distribution of pressure coefficient 
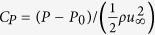
 on the surface of the sphere for Re = 10^5^ and Re = 1.62 × 10^5^ are presented in Fig. 11 and these are compared with the experimental data. It is evident from the plot that the quality of *C*_*P*_ prediction remains very good even with a sphere size of *D* = 160 (lattice points). Even for this size of grid, the error in mean value of C_D_ is only around 8%. Along with this paper, we have also provided a supplementary video 1 online where the temporal evolution of the azimuthal component of vorticity *ω*_*ϕ*_, *C*_*P*_ and *C*_*D*_ are shown. Figure 12 shows a snapshot of the azimuthal component of vorticity. This also shows that our model captures the flow separation clearly. We believe that the reason for such good accuracy without a well-resolved boundary layer is largely due to the fact that the drag for flow past sphere is largely dominated by form drag, which does not crucially depend on boundary-layer resolution. Thus, with moderate resolution requirement, our approach is able to predict quantities of engineering interest quite accurately. This improvement in LBM is remarkable as typical LBM simulations for high Reynolds Numbers for any setup require the use of turbulence models^6^,^47^,^48^,^49^ but present RD3Q27 model does not require any explicit model for turbulence.

## Outlook

A new formulation of LBM, based on the crystallographic viewpoint has been presented. It has been shown that LBM based on the BCC lattice spatial discretization has efficiency that is an order-of-magnitude higher than traditional LBM. The gain obtained by using the BCC lattice has been demonstrated by various representative simulations such as Kida flow and flow past a sphere. To conclude, this new formulation is capable of solving problems of practical interest without using explicit turbulence modeling. Thus, the current approach of formulating LBM on a BCC grid raises the prospect of direct simulations of turbulence without using explicit turbulence models.

Finally, we emphasize that this new route to increase accuracy is not an alternate to building higher order LBM for better accuracy. Indeed by constructing higher order LBM on BCC, accuracy can be further improved^50^. Similarly, role of collision models (multi-relaxation, entropic)^32^,^51^ needs to be investigated for this lattice.

## Additional Information

**How to cite this article**: Namburi, M. *et al*. Crystallographic Lattice Boltzmann Method. *Sci. Rep*. **6**, 27172; doi: 10.1038/srep27172 (2016).

## Supplementary Material

Supplementary Information

Supplementary Video S1

## Figures and Tables

**Table 1:** Energy shells and their corresponding velocities with weights for D3Q27.

Shells	Discrete Velocities (*c*_*i*_)	Weight (*w*_*i*_)
SC	(±1, 0, 0), (0, ±1, 0), (0, 0, ±1)	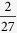
FCC	(±1, ±1, 0), (0, ±1, ±1), (±1, ±1, 0),	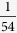
BCC	(±1, ±1, ±1)	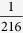

**Table 2:** Energy shells and their corresponding velocities with weights for RD3Q27.

Shells	Discrete Velocities (*c*_*i*_)	Weight (*w*_*i*_)
SC	(±1, 0, 0), (0, ±1, 0), (0, 0, ±1)	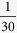
FCC	(±1, ±1, 0), (0, ±1, ±1), (±1, ±1, 0),	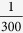
BCC	(±1/2, ±1/2, ±1/2)	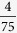

**Table 3:** Comparison of *C*_*D*_ values obtained using the present RD3Q27 model with experimental data^40^.

Re	*C*_*D*_ (expt, Achenbach)	*C*_*D*_ (present)
10^5^	0.4–0.5	0.4957
3.18 × 10^5^	0.453	0.4044
