# Supplementary material for: Crystallographic Lattice Boltzmann Method
Source: Sci Rep. 2016 Jun 1;6:27172. doi: 10.1038/srep27172 (PMC4890317; doi:10.1038/srep27172)
Supplement: Supplementary Information [file srep27172-s1.pdf]

Crystallographic lattice Boltzmann method

Manjusha Namburi, Siddharth Krithivasan, and Santosh Ansumali*
Jawaharlal Nehru Centre for Advanced Scientific Research,
Jakkur, Bangalore 560064, India

* Corresponding author:
ansumali@jncasr.ac.in

SUPPLEMENTARY VIDEO LEGENDS

Supplementary Video 1 Evolution of the azimuthal component of vorticity ω_ϕ for flow past sphere at $\mathrm{Re} = 10^5$. The video shows the temporal variation of vorticity structures (azimuthal component of vorticity) for flow past sphere at $\mathrm{Re} = 10^5$. The comparison of instantaneous coefficient of drag (C_D) and distribution of coefficient of pressure (C_P) on the surface of the sphere with experiments is also shown at the same instant as that of vorticity. It is to be noted that the experimental data is at steady state and the movie shows the instantaneous values of C_D and C_P obtained from the simulations whereas the experimental data is at steady state. The movie depicts the path taken to reach steady state. It can be observed that C_D and C_P compare well with the experimental results and the small scale vorticities are captured.

APPENDIX A

The higher order moments are, $R_{\alpha\beta\gamma\kappa} = \sum_i f_i c_{i\alpha} c_{i\beta} c_{i\gamma} c_{i\kappa}$, $\Phi = \sum_i f_i \left(c_x^4 + c_y^4 + c_z^4\right)$ and $n_\gamma = \sum_i f_i \left(c_x^4 + c_y^4 + c_z^4\right) c_{i\gamma}$.

The closure relations that are necessary to solve the set of simultaneous equations (Eq.14 in the article) can be written as,

$$R_{\alpha\beta\gamma\kappa} = \delta_{\alpha\beta\gamma\kappa\mu\nu}\left(\frac{-43}{15}P_{\mu\nu} + \frac{156}{10}R_{\mu\nu}\right) + \delta^{(4,2)}_{\alpha\beta\gamma\kappa\mu\nu}\left(\frac{7}{5}P_{\mu\nu} - \frac{6}{5}R_{\mu\nu}\right) + \delta_{\alpha\beta\gamma\kappa}\left(\frac{14}{15}P - \frac{3}{10}R - \frac{5}{6}\Phi\right) + \Delta_{\alpha\beta\gamma\kappa}\left(-\frac{2}{3}P + \frac{1}{2}R + \frac{1}{6}\Phi\right) - \frac{2}{5}\left[P_{\alpha\beta}\delta_{\gamma\kappa} + P_{\alpha\gamma}\delta_{\beta\kappa} + P_{\gamma\kappa}\delta_{\alpha\beta} + P_{\gamma\beta}\delta_{\alpha\kappa} + P_{\beta\kappa}\delta_{\gamma\alpha} + P_{\alpha\kappa}\delta_{\beta\gamma}\right] + \frac{1}{5}\left[R_{\alpha\beta}\delta_{\gamma\kappa} + R_{\alpha\gamma}\delta_{\beta\kappa} + R_{\gamma\kappa}\delta_{\alpha\beta} + R_{\gamma\beta}\delta_{\alpha\kappa} + R_{\beta\kappa}\delta_{\gamma\alpha} + R_{\alpha\kappa}\delta_{\beta\gamma}\right], \tag{1}$$

where P=$P_{xx} + P_{yy} + P_{zz}$ and R = $R_{xx} + R_{yy} + R_{zz}$.

$$\begin{pmatrix} N_{xxx} \\ N_{yyy} \\ N_{zzz} \\ N_{xyz} \\ N_{xxy} \\ N_{xxz} \\ N_{yyx} \\ N_{yyz} \\ N_{zzx} \\ N_{zzy} \end{pmatrix} = \begin{pmatrix} -\frac{3j_x}{4} + \frac{7Q_{xxx}}{4} + Q_{yyx} + Q_{zzx} \\ -\frac{3j_y}{4} + Q_{xxy} + \frac{7Q_{yyy}}{4} + Q_{zzy} \\ -\frac{3j_z}{4} + Q_{xxz} + Q_{yyz} + \frac{7Q_{zzz}}{4} \\ \frac{3Q_{xyz}}{4} \\ \frac{1}{12}(-5j_y + 24Q_{xxy} + 5Q_{yyy}) \\ \frac{1}{12}(-5j_z + 24Q_{xxz} + 5Q_{zzz}) \\ \frac{1}{12}(-5j_x + 5Q_{xxx} + 24Q_{yyx}) \\ \frac{1}{12}(-5j_z + 24Q_{yyz} + 5Q_{zzz}) \\ \frac{1}{12}(-5j_x + 5Q_{xxx} + 24Q_{zzx}) \\ \frac{1}{12}(-5j_y + 5Q_{yyy} + 24Q_{zzy}) \end{pmatrix},$$

$$\begin{pmatrix} n_x \\ n_y \\ n_z \end{pmatrix} = \begin{pmatrix} -\frac{3j_x}{4} + \frac{7Q_{xxx}}{4} + Q_{yyx} + Q_{zzx} \\ -\frac{3j_y}{4} + Q_{xxy} + \frac{7Q_{yyy}}{4} + Q_{zzy} \\ -\frac{3j_z}{4} + Q_{xxz} + Q_{yyz} + \frac{7Q_{zzz}}{4} \end{pmatrix}.$$
